# *Helicobacter pylori* Initiates a Mesenchymal Transition through ZEB1 in Gastric Epithelial Cells

**DOI:** 10.1371/journal.pone.0060315

**Published:** 2013-04-02

**Authors:** Jessica Baud, Christine Varon, Sandrine Chabas, Lucie Chambonnier, Fabien Darfeuille, Cathy Staedel

**Affiliations:** 1 University Bordeaux, ARNA Laboratory, Bordeaux, France; 2 INSERM, U869, ARNA Laboratory, Bordeaux, France; 3 University Bordeaux, Laboratoire de Bactériologie, Bordeaux, France; 4 INSERM, U853, Laboratoire de Bactériologie, Bordeaux, France; Veterans Affairs Medical Center (111D), United States of America

## Abstract

Chronic *Helicobacter pylori* infection provokes an inflammation of the gastric mucosa, at high risk for ulcer and cancer development. The most virulent strains harbor the *cag* pathogenicity island (*cag*PAI) encoding a type 4 secretion system, which allows delivery of bacterial effectors into gastric epithelial cells, inducing pro-inflammatory responses and phenotypic alterations reminiscent of an epithelial-to-mesenchymal transition (EMT). This study characterizes EMT features in *H. pylori*-infected gastric epithelial cells, and investigates their relationship with NF-κB activation. Cultured human gastric epithelial cell lines were challenged with a *cag*PAI*+ H. pylori* strain or *cag* isogenic mutants. Morphological changes, epithelial and mesenchymal gene expression and EMT-related microRNAs were studied. *H. pylori* up-regulates mesenchymal markers, including ZEB1. This transcription factor is prominently involved in the mesenchymal transition of infected cells and its up-regulation depends on *cag*PAI and NF-κB activation. ZEB1 expression and NF-κB activation were confirmed by immunohistochemistry in gastric mucosa from *cag*PAI*+ H. pylori*-infected patients. Gastric epithelial cell lines express high miR-200 levels, which are linked to ZEB1 in a reciprocal negative feedback loop and maintain their epithelial phenotype in non-infected conditions. However, miR-200b/c were increased upon infection, despite ZEB1 up-regulation and mesenchymal morphology. In the miR-200b-200a-429 cluster promoter, we identified a functional NF-κB binding site, recruiting NF-κB upon infection and trans-activating the microRNA cluster transcription. In conclusion, in gastric epithelial cells, *cag*PAI+ *H. pylori* activates NF-κB, which transactivates ZEB1, subsequently promoting mesenchymal transition. The unexpected N-FκB-dependent increase of miR-200 levels likely thwarts the irreversible loss of epithelial identity in that critical situation.

## Introduction

The bacterium *Helicobacter pylori*, which colonizes the stomach of 50% of the world population, arouses a chronic inflammation in the gastric mucosa and constitutes the greatest risk factor for gastric cancer. The severity of the inflammation and the gastric malignant progression are strongly associated to the presence of the *cag* pathogenicity island (*cag*PAI) in the bacterial genome [1]. *H. pylori cag*PAI encodes a type 4 secretion system (T4SS) and the effector protein CagA [2]. CagA and the bacterial peptidoglycan (PG) are translocated *via* the T4SS into gastric epithelial cells, and subsequently trigger cell innate immunity by activating the nuclear factor κB (NF-κB), a master transcription factor in inflammatory responses following microbial infection [3]. NF-κB activation is mediated by CagA interactions with TAK1 and TRAF6 and PG recognition by NOD1, and leads to NF-κB-dependent transcription of multiple target genes, including the pro-inflammatory interleukin (IL)-1β, IL-6, IL-8 and tumor necrosis factor α [4]–[6]. Besides, CagA binds to numbers of other host proteins involved in cell junctions and signal transductions, activating ERK-MAPK, ß-catenin and c-Met signaling pathway and impairing epithelial cell polarity [7]–[12].

In some gastric epithelial cell lines such as AGS, a cell culture model widely used to recapitulate early events of *H. pylori* infection, *cag*PAI*+* strains induce a characteristic morphological transformation termed the hummingbird phenotype, an extremely elongated cell shape along with loss of cell-cell contacts. The relevance for the hummingbird phenotype in gastric carcinogenesis has been provided by the observation that *H. pylori* strains isolated from gastric carcinoma tissues, in majority *cag*PAI*+*, are indeed able to induce this phenotype [13]; [14]. Induction of the hummingbird phenotype resembles epithelial to mesenchymal transition (EMT), a reversible tissue remodeling process, during which epithelial cells lose cell-cell contacts and acquire a spindle-like morphology. EMT occurs during development, wound healing, and epithelia-derived tumor invasion and metastasis, and is underlain by specific changes in gene expression that regulate the mesenchymal versus epithelial phenotype [15]; [16]. The zinc finger E-box-binding homeobox 1 and 2 (ZEB1/2) transcription factors are crucial EMT activators repressing epithelial-specific *cdh1, crumbs-3 and lgl-2* genes [17]–[19]. ZEB1/2 are reciprocally linked to the miR-200 family members in a negative feedback loop, each strictly regulating the expression of the other, thereby controlling both the stability and reversibility of the epithelial versus mesenchymal phenotypes [16]; [20]. MiR-200 are microRNA (miRNA), small noncoding RNA molecules that post-transcriptionaly regulate gene expression in a variety of biological process [21]. The miR-200 are produced from two miRNA clusters, *miR-200b-200a-429* and *miR-200c-141*, the promoters of which harbor ZEB1/2 binding sites and are repressed by ZEB1/2 [22]; [23]. In turn, miR-200 post-transcriptionaly silence ZEB1/2 by inhibiting translation of their mRNA.

We previously reported that, while activating NF-κB and inducing the hummingbird phenotype, *H. pylori* blocked cell cycle progression in a CagA-dependent manner, post-transcriptionaly de-repressing the Large Tumor Suppressor 2 (LATS2) upon miR-372-373 down-regulation [24]. In the present study we address the question whether *cag*PAI*-*dependent NF-κB activation and EMT-like cell elongation may be connected, meaning that gastric epithelial cells undergo an EMT in the context of *H. pylori*-elicited inflammation. First, we report that *H. pylori* up-regulates mesenchymal genes, among which ZEB1 is prominently involved in the mesenchymal morphology of the infected gastric epithelial cells. Next, we analyzed changes in miR-200b and -200c levels upon infection. At last, we demonstrated that ZEB1 and miR-200 were both dependent on *cag*PAI, which activates NF-κB. We propose a mechanism whereby *cag*PAI*+ H. pylori*-sustained NF-κB activation transactivates both positive and negative EMT regulators. Collectively, these findings demonstrate the existence of NF-κB/ZEB/miR-200 signaling network that initiates mesenchymal transition of *H. pylori*-infected gastric cells.

## Materials and Methods

### *Helicobacter pylori* Culture

*H. pylori* 26695 strain (CIP 106780, Institut Pasteur, Paris, France) and its isogenic knockout mutants Δ*cagA* and Δ*cagE* were grown on columbia agar plates as previously described [24].

#### Cell culture

AGS (ATCC CRL-1739) and NCI-N87 (ATCC CRL-5822) cells were grown in DMEM/Ham F-12 medium, MKN-74 (Health Science Research Resources Bank JCRB0255) in RPMI medium, and HeLa (ECCAC 93021013) and HEK293 (ECCAC 85120602) in DMEM. All media were supplemented with 10% heat-inactivated fetal bovine serum and 2 mM L-glutamine (all from Invitrogen, France). All co-culture experiments with *H. pylori* were performed as previously described at a multiplicity of infection (MOI) of 100 for 24 hours at 37°C, in a humidified 5% CO_2_ atmosphere [24].

### RNA Extraction and Quantitative RT-PCR

RNA extractions and RT-qPCR experiments on mRNA, miRNA and pri-miRNA were performed as described previously and in Material & Methods S1 (specific primers are listed in Table S1) [24].

### Western Blot

Cells were lysed and proteins were extracted and submitted to SDS-PAGE and western blot for immunostaining of ZEB1 (1∶500 dilution, Bethyl) and α-tubulin (1∶30,000 dilution, Sigma-Aldrich) and chemoluminescent detection as previously described [24].

### Transfections and Reporter Assays

All transfections experiments of oligonucleotides or expression vectors were performed in 24-well plates using Lipofectamine 2000 (Invitrogen) according to the manufacturer’s protocol. See Material & Methods S1 for detailed informations on vector construction, transfections, and expression of results of reporter assays.

### Immunofluorescence

Cells were grown on glass coverslips, fixed with 3% buffered paraformaldehyde and permeabilized in Triton 0,2%. After blocking with 3% bovine serum albumine in PBS, they were stepwise incubated overnight at 8°C with either a rabbit anti-ZEB1 antibody (1∶400 dilution) or a goat anti-p65 NF-κB antibody (1∶100 dilution; Tebu, France), and then 30 min at room temperature with a secondary anti-rabbit IgG antibody coupled to Alexafluor488 or a secondary anti-goat IgG antibody couplet to Alexafluor 564 (1∶2,000 dilution; Invitrogen). The coverslips were mounted on glass slides using Slowfade reagent (Invitrogen) prior to imaging in epifluorescence on a Zeiss microscope.

### Human Gastric Tissue Immunohistochemistry and *in situ* Hybridization of miR-200b

This work has been approved by the Direction for Clinical Research of the University Hospital Centre of Bordeaux (France). Considering that the work has been performed on surgical waste (for *Helicobacter* culture and identification, and immunochemistry analyses), but not on tissue samples specifically collected for research purpose, approval from the Committee for Person Protection (CPP South-West, France) was not required. Gastric fresh tissue samples have been collected by pathologists in healthy area distant from the tumor site, from consenting patients (written consent), who underwent gastrectomy for gastric cancer, in agreement with the Tumor and Cell Bank of the University Hospital Centre of Bordeaux (France). Only tissue samples that were initially taken for diagnostic purposes were secondarily used for the present study and were de-identified and considered as samples normally discarded. See material & Methods S1 for histology processing, immunohistochemistry, *in situ* hybridization of miR-200b and *Helicobacter* detection. Only *cag*PAI+ strains have been isolated from the gastric tissues of our collection.

### Chromatin Immunoprecipitation Assays

The complete protocol is detailed in Material & Methods S1.

## Results

### *H. pylori* Up-regulates ZEB1 in Gastric Epithelial Cells

Upon 24 h in presence of a *cag*PAI*+ H. pylori* strain (wild type, wt), human gastric epithelial cell lines undergo morphological changes (Fig. S1). AGS cells become drastically elongated with loosen intercellular junctions (the hummingbird phenotype); elongation affects 77.9±19.4% of cells upon infection (versus 1% in the non infected cell population, n = 6, p<0.01). In the infected NCI-N87 cell line, cell elongation is only visible at the border of the colonies, which are very dense and unsuitable for numeration. MKN-74 cells detach from the cell layer in small clusters of living cells, leaving the cell monolayer quite unchanged upon infection. Whatever the cell line, these alterations are not visible with CagA-deleted (Δ*cagA*) or T4SS-impaired (Δ*cagE*) isogenic *H. pylori* mutants (Fig. S1). This suggests that gastric epithelial cells challenged with wt *H. pylori* initiate a mesenchymal phenotype.

In order to further characterize the phenotype induced by wt *H. pylori*, we investigated quantitative changes in several mesenchymal and epithelial gene expressions (Table 1). We observe a global up-regulation of mesenchymal markers, including ZEB1, Vimentin, BMP1, Snail1, Snail3, Integrin α5 and MMP9, along with down-regulation of some epithelial markers such as Keratin 7 and Osteopontin (SPP1), except E-Cadherin, that we find enhanced up to 7 fold in NCI-N87 cells. As E-cadherin down-regulation is the hallmark of EMT, we further investigated this discrepancy by analyzing its expression and localization by immunofluorescence. Whereas E-cadherin is detectable at the cell membrane of non infected cells as a component of the adherens junction complexes, its appears disorganized and punctiform in the cytoplasm of infected NCI-N87 cells (Fig. S2). This altered location suggests a loss of function of E-cadherin as cell-cell adhesion molecule, despite the apparent cohesiveness of the cell layer (Fig. S1) and the increase of its mRNA upon infection (Table 1). AGS cells do not express E-cadherin protein, although its mRNA is detectable: this absence likely may contribute to the high plasticity of this cells line, as compared to the two others [11].

**Table 1:** Changes in expression of mesenchymal and epithelial markers in AGS, MKN74 and NCI-N87 cells infected for 24 h with the H. pylori strain 26695.

		Fold changes induced by *cagPAI+ H. pylori*		
		AGS	MKN74	NCI-N87
**Mesenchymal markers**	**BMP1**	2.46*	1.78	2.11
	**ITGA5**	3.08	11.19**	3.19
	**MMP9**	5.08***	14.78*	1.73
	**Slug**	Not detected	3.68	3.34
	**Snail 1**	2.64**	4.64*	1.24
	**Snail 3**	2.22*	3.43	4.51
	**Twist**	Not detected	Not detected	1.3
	**Vimentin**	1.2*	2.38**	2.42**
	**ZEB1**	4.3**	19.26***	3.56*
	**ZEB2**	Not detected	Not detected	Not detected
**Epithelial markers**	**KRT7**	− 2.03***	−2.17	2.18
	**SSP1**	−4.69**	2.2	Not detected
	**E-cadherin**	2.01*	2.1	7.07

Data represent RT-qPCR results of the individual genes normalized to that of HPRT and compared to non infected cells (mean, n = 3, *p<0.05, **p<0.01, ***p<0.001).

**Figure 1:**
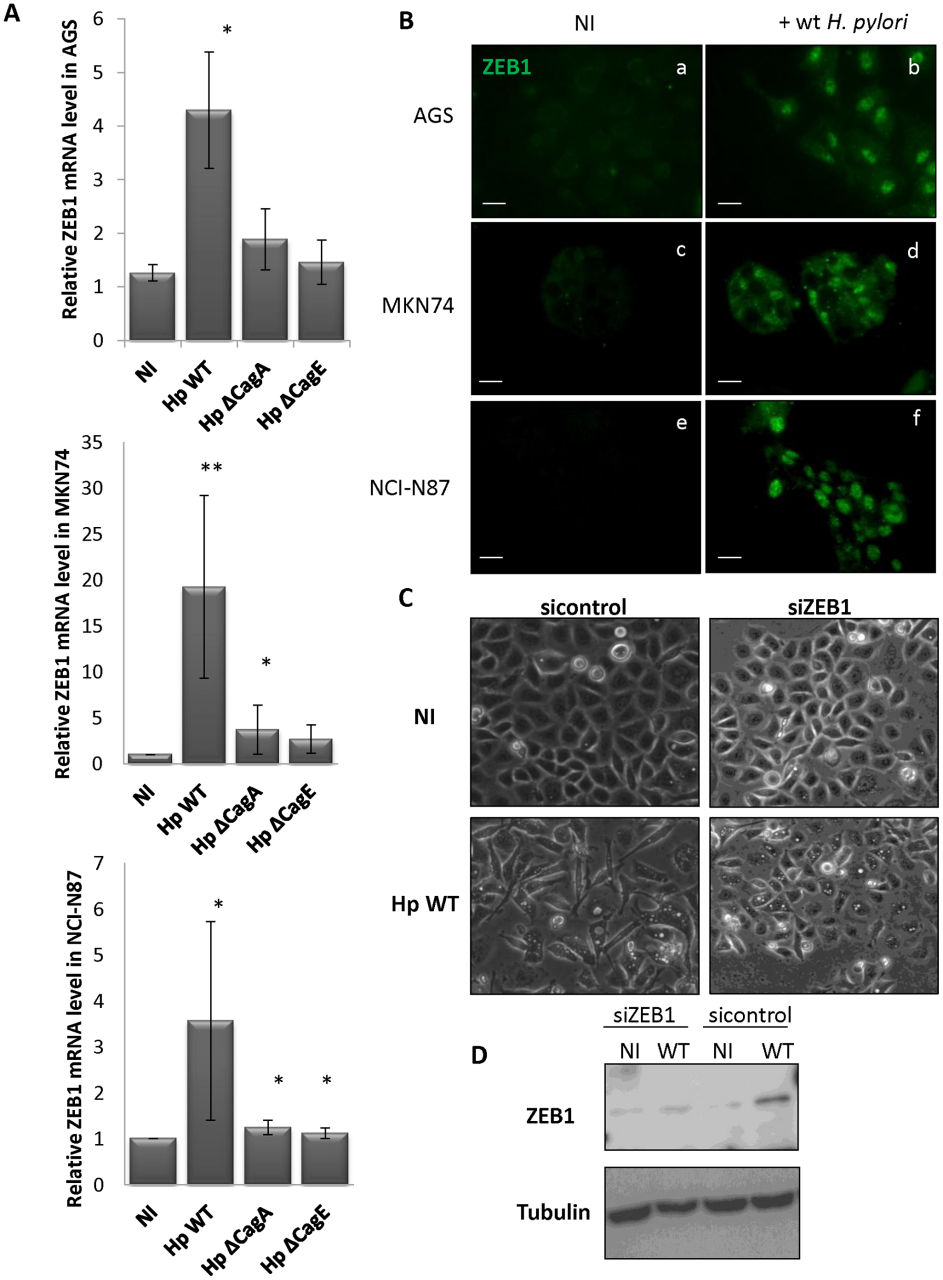
*H. pylori* up-regulates ZEB1 in gastric epithelial cells. (A) RT-qPCR data of ZEB1 mRNA upon 24 h infection with wild type *H. pylori* (Hp WT) or its isogenic mutants deleted either for *cagA* (Hp Δ*cag*A) or *cagE* (Hp Δ*cag*E); bars represent the mean ± SD of ZEB1 mRNA relative to HPRT mRNA compared to non infected cells (NI) (n = 5; * p<0.05). (B) ZEB1 immunofluorescence in non-infected cells (NI) or upon infection as in (A); bar, 40 µm. (C) Cell morphology observed by phase contrast microscopy of AGS cells transfected with siZEB1 or control siRNA (20 nM) prior infection as in (A). Bar, 40 µm. (D) ZEB1 or α-tubulin immunoblots in cells treated with siZEB1 or control siRNA and infected as in (A).

In every cell line, ZEB1 mRNA is significantly enhanced in presence of wt *H. pylori,* but quite unaffected with Δ*cag*A or Δ*cag*E mutants (Fig. 1A). ZEB1 protein accumulates in the nucleus upon infection (Fig. 1B), in agreement with its function as transcription factor. In order to assess ZEB1 importance in these morphological changes, we took advantage of the AGS cell line for its obvious mesenchymal shape induced by wt *H. pylori* strain (Fig. S1). Treating the cells with anti-ZEB1 siRNA (siZEB1) before infection prevents both ZEB1 induction (Fig. 1D) and mesenchymal morphology (Fig. 1C), contrarily to control siRNA (siControl). Indeed, only 8.12±1.74% siZEB1-treated, infected cells adopted the hummingbird phenotype (versus 72.81±33.32% siControl-treated, infected cells, n = 5, p<0.01). All together, these data suggest that in gastric epithelial cell lines, *H. pylori* initiates a *cagPAI-*dependent mesenchymal transition, in which ZEB1 plays a major role.

### ZEB1 is Repressed by miR-200 in Gastric Epithelial Cells

**Figure 2:**
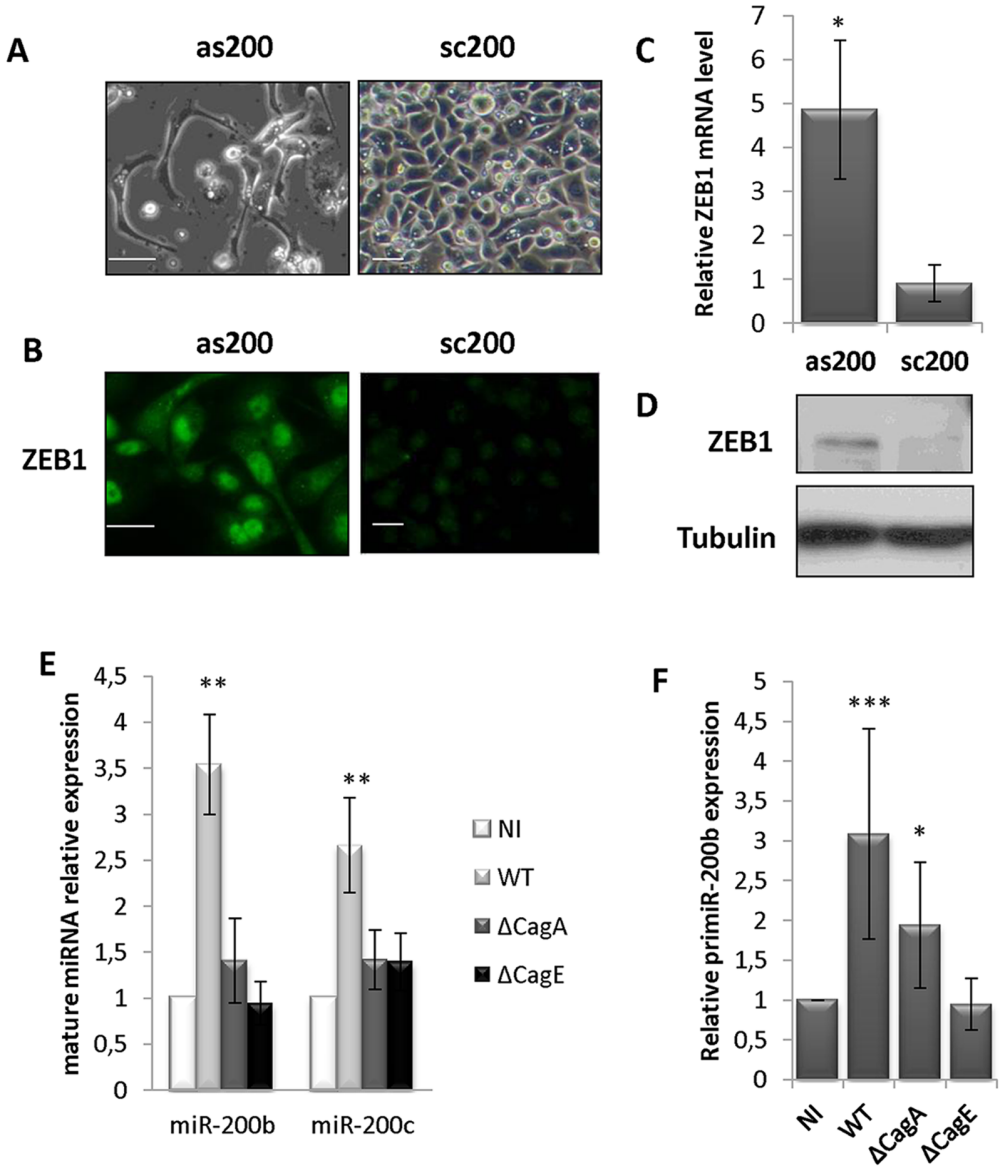
MiR-200 regulate ZEB1 expression in basal conditions and are up-regulated by *H. pylori*. (A) Cell morphology observed by phase contrast microscopy of AGS cells treated for 5 day with 100 nM antisense (as200b/c) or scrambled (sc200) oligonucleotides (scale bar, 20 µm). (B) ZEB1 immunofluorescence in the same conditions (scale bar, 20 µm) (C) RT-qPCR data of ZEB1 mRNA in the same conditions. Bars represent the mean ± SD of ZEB1 mRNA relative to HPRT mRNA compared to non transfected cells (n = 5; * p<0.05). (D) ZEB1 and tubulin immunoblots in the same conditions. (E) RT-qPCR data of mature miR-200b and -200c in AGS cells infected or not (NI) with either wt *H. pylori* (WT) or isogenic mutants (ΔCagA, ΔCagE) at MOI 100 bacteria/cells for 24 h. Bars indicate mean ± SD of miRNA expression normalized to U6 snRNA and compared to NI (n = 6; ** p<0.01). (F) RT-qPCR data of primary miR-200b-200a-429 transcript in the same infection conditions. Bars indicate mean ± SD of pri-miRNA expression normalized to HPRT1 and compared to NI (n = 4; * p<0.05, *** p<0.01).

Our previous data on AGS miRNA profile indicated that these cells express mainly miR-200b and miR-200c of that family, which share a quite similar sequence [24]. This result was confirmed in the three gastric cell lines in Table S2A. ZEB1 expression is inversely correlated to that of miR-200 in the different cell lines tested, with high miR-200b and -200c and low ZEB1 expressions in the human gastric epithelial cell lines, and low miR-200b and -200c and high ZEB1 expressions in HeLa or HEK293 cells (Fig. S3A), accordingly to the reciprocal negative feedback loop linking ZEB1 and the miR-200. Whereas scrambled control oligonucleotide (sc200) affect neither miR-200 levels (Fig. S3B), nor the original cobblestone cell morphology, antisense oligonucleotides (as200b, as200c) specifically decreases by 75% the detectable miR-200b and miR-200c levels (Fig. S3B) and dramatically affects cell morphology, which turns for 62.88±28.53% cells (versus 3.44±1.85% in sc200-treated cells, n = 9, p<0.001) in an extremely elongated shape similar to the one provoked by *H. pylori* (Fig. 2A). In MKN-74 cells, as200b/c treatment causes the formation of floating clusters of living cells similar to those represented in Fig. S1 in presence of *H. pylori* (data not shown). This experiment could not be realized in NCI-N87, which are poorly transfectable. As200b/c specifically up-regulate ZEB1 mRNA and protein levels (Fig. 2C and D, respectively), and ZEB1 accumulates in the nucleus (Fig. 2B). To assess whether ZEB1 is regulated at a post-transcriptional level by the endogenous miR-200b/200c levels, we evaluated ZEB1 translation efficiency using the psiZEB1 luciferase sensor containing ZEB1 3′UTR, which harbors 8 miR-200b/200c pairing sites, downstream to *Renilla* luciferase coding sequence (Fig. S3C). Luciferase activity generated by psiZEB1 is repressed by 50% as compared to the control vector and totally de-repressed in as200b/c-treated cells, but not in sc200-treated cells (Fig. S3D), correspondingly to the post-transcriptional regulation of ZEB1 by endogenous miR-200b/c levels.

### *H. pylori* Up-regulates miR-200b/c

The above data show that the mesenchymal morphology of AGS cells driven either by *H. pylori* or miR-200b/c loss of function is involving ZEB1 up-regulation, and suggest that miR-200b/c down-regulation could also participate to ZEB1 induction upon infection. This prompted us to analyze changes in miR-200b/c levels upon infection. Unexpectedly, we observe a significantly enhanced expression of both miR-200b and miR-200c by wt *H. pylori,* but not by the Δ*cag*A or Δ*cag*E mutants (Fig. 2E). *Cag*PAI*-*dependent miR-200b and -200c up-regulations are also observed in infected MKN-74 and NCI-N87 cells (Fig. S4). The other miR-200 family members miR-200a, -141 or -429 are diversely affected by the infection (Table S2B). In AGS and MKN74 cells, miR-429, which shares the same seed sequence than miR-200b and -200c, is likewise up-regulated upon infection with wt *H. pylori*. In NCI-N87 cells, miR-200a and miR-141, which belongs to the same clusters than miR-200b and miR-200c, respectively, are also positively affected by infection. However, the contributions of miR-200a, -141 and 429 to the maintenance of the epithelial phenotype of gastric cells may likely be minimal compared to those of miR-200b and miR-200c, because of their much lower abundance.

**Figure 3:**
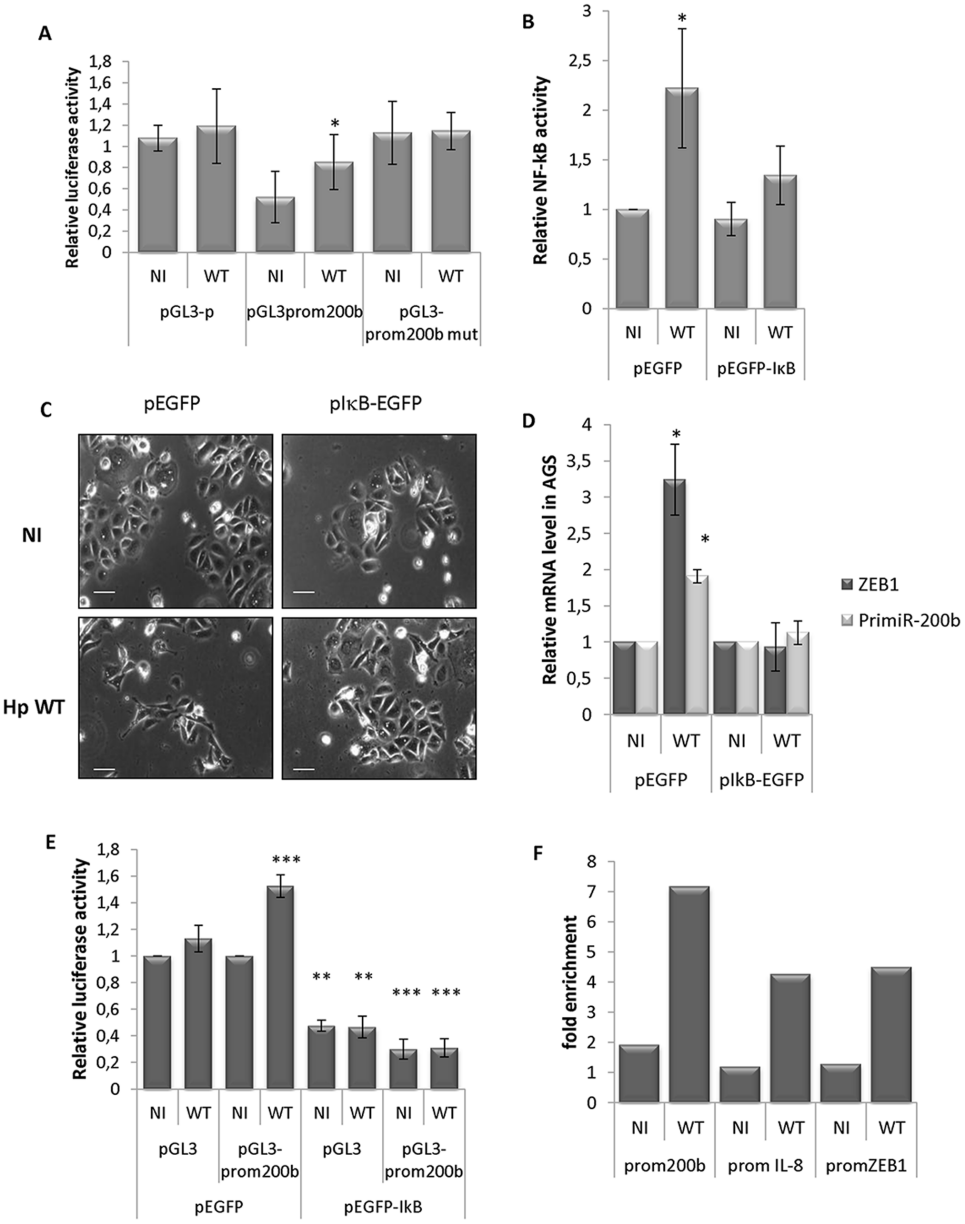
NF-κB-dependent mesenchymal phenotype of infected AGS cells. In all experiments, AGS cells were infected or not (NI) with *cagPAI+ H. pylori* (WT) at MOI 100 bacteria/cells for 24 h. (A) Activities of SV40 promoter (pGL3-p), or *miR-200b-200a-429* promoter wild type (pGL3-prom200b) or mutated on the NF-κB site (pGL3-prom200b mut); bars represent mean ± SD of relative luciferase activities of each promoter reporter normalized to that of NI pGL3-p transfected cells (n = 3; ** p<0.01). (B) NF-κB activation upon infection (WT) in cells transfected either with pEGFP or with pEGFP-IκB. Bars represent mean ± SD of relative NF-κB reporter luciferase activity compared to NI pEGFP-transfected cells (n = 3; *p<0.05). (C) Cell morphology observed by phase contrast microscopy, in the same conditions. Bar, 20 µm. (D) RT-qPCR data of ZEB1 and pri-miR-200b-200a-429 in pEGFP- or pEGFP-IκB-transfected cells. Bars indicate mean ± SD of RNA expression normalized to HPRT1 and compared to NI (n = 3, * p<0.05, *** p<0.001). (E) *MiR-200b-200a-429* promoter activities measured as in (A) in cells transfected either with pEGFP or with pEGFP-IκB. (F) Chromatin immunoprecipitation assays using anti-NF-κB antibody on the promoters of miR-200b (prom200b), IL-8 (promIL-8) or ZEB1 (promZEB1). Bars represent NF-κB enrichment on a given promoter in either uninfected or infected cells, calculated as the following ratio: 2^−ΔCt IPNF-κB^/2^−ΔCt controlIP^, with ΔCt = Ct IP (with NF-κB antibody or without (control)) – Ct input (input corresponds to chromatin before immunoprecipitation).

In parallel to enhanced mature miRNA levels, up-regulation of the primary miR-200b-200a-429 transcript leading to mature miR-200b production is likewise dependent on *H. pylori cag*PAI (Fig. 2F), since the isogenic mutants deleted for either *cag*A or *cag*E are twice less or not efficient, respectively, for enhancing pri-miR-200b levels. This suggests that the enhanced mature miR-200b levels may result from enhanced transcription of its gene cluster in a *cag*PAI-dependent manner. To confirm this hypothesis, we analyzed the activity of the *miR-200b-200a-429* promoter using the pGL3-prom200b luciferase reporter driven by the 461 base pairs minimal promoter of that cluster, which contains the regulatory elements responsible of epithelial-specific expression [22]. This promoter region produces a noticeable luciferase activity reaching 50 and 80% of that of the SV40 promoter in AGS and MKN-74 cells, respectively, but not in HEK293 cells (Fig. S5A), in direct relationship with miR-200b levels and inverse relationship with their transcriptional repressor ZEB1 (Fig. S3A). However, despite ZEB1 induction by *H. pylori*, the activity of the miR-200b promoter is stimulated in infected AGS (Fig. 3A), as well as in infected MKN-74 cells (Fig. S5B), whereas the SV40 promoter of the pGL3-p reporter remains insensitive to the bacteria. This result excludes that activation of the miR-200b promoter results from enhanced general transcriptional activities in infected cells, and confirms that miR-200b biosynthesis was specifically stimulated upon infection.

### ZEB1 and miR-200b Up-regulation Both Depend on NF-κB Activation

In order to understand the mechanism whereby *H. pylori* paradoxically up-regulates miR-200b, we searched putative binding sites for transcription factors within the *pri-miR-200b-200a-429* promoter region, in addition to the paired E-boxes to which ZEB1 must bind to achieve transcriptional repression [22]. We found a putative NF-κB binding site overlapping one of the E-box (Fig. S5C). In order to demonstrate that this site is functional, we mutated it at a position at which it may not affect the E-box. Compared to the parental *H. pylori-*stimulated miR-200b promoter activity, the mutated promoter becomes insensitive to the bacteria (Fig. 3A). However, its basal activity is raised at the level of that of the pGL3-p SV40 promoter. Our pGL3-prom200b mutated for NF-κB binding site behaves as the one mutated for the E-Box described by Braken *et al*. [22]. This suggests that the mutations we performed could also have affected the ZEB1 binding site. The putative binding sites for general transcription factors such as Sp1 and AP-2 (Fig. S5C) could contribute positively to the promoter activity.

Interestingly, a NF-κB binding site has been previously characterized on the *zeb1* promoter [25]. Combined to our miR-200b promoter analysis, this points NF-κB as a common transactivator of *zeb1* and *primiR-200b-200a-429*, along with the pro-inflammatory IL-1β, IL-6, or IL-8 genes. To assess whether the mesenchymal transition of *H. pylori*-infected gastric epithelial cells may be driven by NF-κB, we prevented NF-κB activation by overexpressing IκB with an EGFP-IκB expression vector transiently transfected prior infection (Fig. 3B–D). In basal conditions, NF-κB immunostaining is positive in the cytoplasm and excludes the nucleus (Fig. S6, upper panels). Upon infection, the activated NF-κB is translocated into the nucleus of control EGFP-transfected cells, so that NF-κB immunostaining is positive in the whole cell including the nucleus (Fig. S6, left lower panel); this translocation is prevented in EGFP-IκB-transfected cells, in which NF-κB stays in the cytoplasm like in uninfected cells (Fig. S6, right lower pannel). Whereas 43.61±15.84% control EGFP-transfected AGS cells switch to a mesenchymal shape in presence of *H. pylori* (Fig. 3C), most IκB-transfected cells conserve their epithelial morphology upon infection (Fig. 3C), with only 8.83±4.76 adopting the hummingbird phenotype (n = 6, p<0.01). Preventing NF-κB activation (Fig. 3B), IκB abolishes ZEB1 up-regulation in infected cells (Fig. 3D). Moreover, it abolishes miR-200b promoter activity in both basal and *H. pylori*-stimulated conditions (Fig. 3E) and subsequently pri-miR-200b-200a-429 overproduction (Fig. 3D). The IκB-related inhibition of the miR-200b promoter activity may be partly due to the inhibition of general transcription activity, since pGL3-p vector activity is also reduced in IκB-transfected cells as compared to control cells (Fig. 3D). At last, chromatin immunoprecipitation (ChIP) assays shows that AGS cells recruit 3 to 4 fold more endogenous NF-κB onto miR-200b, ZEB1 and IL-8 promoters upon wt *H. pylori* infection (Fig. 3F), indicating that NF-κB directly binds on each of these promoters.

These combined results indicate that 1) the ZEB1-mediated mesenchymal transition of infected AGS cells depends on NF-κB activation and 2) while stimulating this EMT inducer, NF-κB is also responsible for enhanced expression of the EMT negative regulator miR-200b. However, the effects of miR-200 overproduction seem to be dominated by those of ZEB1, since infected cells nevertheless undergo an EMT.

### Kinetics of Changes in Epithelial and Mesenchymal Marker Expression Upon Infection

**Figure 4:**
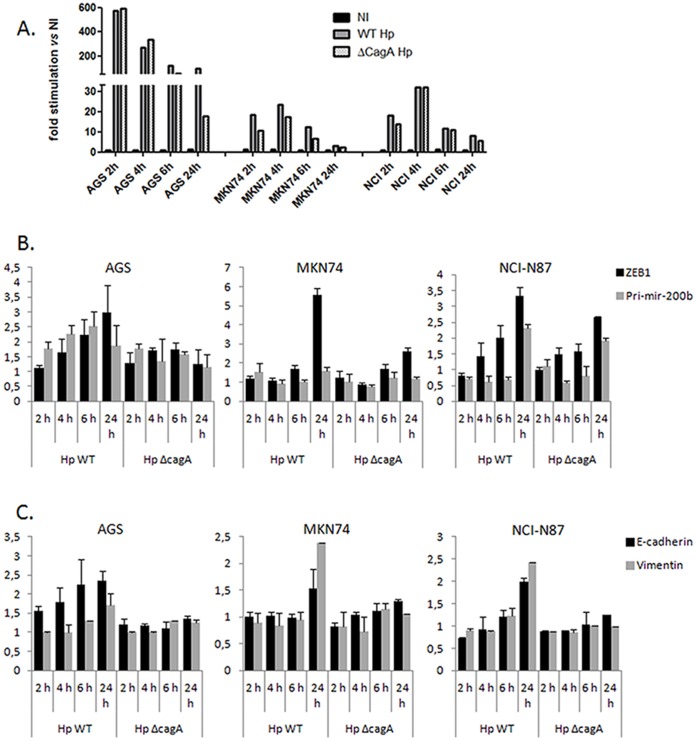
Kinetics of changes in IL-8 induction (A), ZEB1 and pri-miR-200b (B) and E-cadherin and vimentin (C) expressions upon infection. Cells were infected with either wt *H. pylori* (WT) or its *cagA*-deletion isogenic mutant (ΔCagA) at MOI 100 bacteria/cells for the indicated period of times. Bars indicate the fold changes of the individual genes upon infection (mean of duplicates ± SD of RNA expression normalized to HPRT1 and compared to NI).

To assess how these NF-κB targets are orchestrated during infection, we analyzed the time-course of their expressions in the gastric epithelial cells challenged by either wt or *cagA*-deficient *H. pylori*. In every cell line, IL-8 induction is a very early event upon infection, which is maximal during the 2 to 4 hours in the presence of the bacteria and independent of CagA (Fig. 4A), likely involving *H. pylori* PG recognition by NOD1 [3]. IL-8 induction declines thereafter and becomes CagA-dependent at 24 h post-infection. In AGS cells, both the pri-miR-200b RNA and ZEB1 mRNA start rising during the 2 to 4 hrs post-infection in a quite CagA-independent way, thus paralleling IL-8 changes (Fig. 4B). In this early period, E-cadherin is also up-regulated, but its changes depend on CagA (Fig. 4C). All the markers, including vimentin, progressively increase thereafter in presence of the wt bacteria, but not the *cagA*-deleted mutant, while the IL-8 response remains still high. In MKN74 cells, ZEB1 starts rising at 6 hours post-infection independently on CagA, and the pri-miR-200b, E-cadherin and vimentin up-regulations were noticeable only at 24 hrs post-infection with the wt bacteria (Fig. 4 B and C). In NCI-N87 cells, ZEB1 expression starts increasing at 4 hrs upon infection and quite independently of CagA. Note worthily, the pri-miR-200b appears then to be transiently down-regulated by 30–40% (Fig. 4B). Finally, at 24 hrs post-infection, all the markers were up-regulated: the pri-miR-200b and ZEB1, along with IL-8, by both wt and the CagA-deleted strain, E-cadherin and vimentin by the wt bacteria only. Thus, while the activation of the NFκB-ZEB1-miR-200 axis and the concomitant up-regulations of both epithelial and mesenchymal markers upon 24 hr infection is a common feature of the three gastric cell lines, each of them displays specific kinetics in the early time points of infection, likely dependent on their genetic background and their sensitivity to the pathogen. The kinetics studies emphasize the temporal relationship between IL-8, ZEB1 and pri-miR-200b changes, all occurring in the first hours of co-culture and likely mediated by the early NFκB activation upon detection of the microbial virulence factors. On the contrary, E-cadherin and vimentin, the variations of which exhibit different kinetics and are dependent on CagA, may be regulated by another signaling pathway.

The reversibility of these changes has been assessed by sub-culturing the cells post-infection. This experimental procedure likely removes most of the bacteria, as there was no more evidence of residual bacteria after one week. In addition, this extended tissue culture period is unsuitable for the growth of the micro-aerophilic bacteria. As shown in Table 2, the expression levels of IL-8 and ZEB1 in the cells that have been infected are restored to those of non infected cells, whereas vimentin and E-cadherin levels show a trend to be decreased in AGS and MKN74 cells, respectively. This is also the case of the pri-miR-200b RNA in AGS cells. The mature miR-200b and -200c levels were significantly decreased in AGS and NCI-N87 cells, suggesting that these cell lines keep a track of their previous infection despite the apparent reversibility of ZEB1 up-regulation.

**Table 2:** Changes in mesenchymal and epithelial gene expression and miR-200 levels. 10 days post-infection with cagPAI+ H. pylori (Hp WT) or the isogenic CagA-deficient strain.

	Fold changes upon 10 days infection by *H. pylori*					
	AGS		MKN74		NCI-N87	
	WT	Δ*cagA*	WT	Δ*cagA*	WT	Δ*cagA*
**IL-8**	0.678±0.112	0.821±0.019	1.626±0.157	0.93±0.08	0.883±0.091	1.037±0.270
**ZEB1**	1.153±0.535	1.148±0.234	1.297±0.501	1.529±0.644	0.944±0.388	0.973±0.599
**Vimentin**	0.607±0.107	0.597±0.012	0.930±0.045	0.655±0.034	0.809±0.069	0.758±0.047
**E-cadherin**	0.937±0.049	1.171±0.487	0.731±0.256	0.623±0.106	0.813±0.263	0.925±0.243
**Pri-miR-200b**	0.659±0.108	0.937±0.563	0.959±0.544	0.723±0.113	0.867±0.408	0.899±0.383
**miR-200b**	0.587±0.096 (**)	0.663±0.080 (**)	0.969±0.256	0.894±0.102	0.818±0.044 (***)	0.981±0.077
**miR-200c**	0.592±0.050 (**)	0.852±0.172	0.971±0.096	0.876±0.029	0.564±0.098 (***)	0.723±0.189 (*)

48 hrs post-infection at a MOI 100, infected and non-infected cells were trypsinized and subcultured for 10 days in a 6-well plate starting at an initial cell density of 2,000 cells/well. The culture medium was changed every other day. Data represent mean ± SD of RTqPCR results of the individual genes or miRNAs relative to HPRT1 or snoR25, respectively, and compared to non infected cells; n = 4; *: p-value <0.05, **: p-value <0.01, ***: p-value <0.001.

### ZEB1 and miR-200 are Overexpressed in Human Gastric Mucosa in the Context of *H. pylori*-driven Inflammation

**Figure 5:**
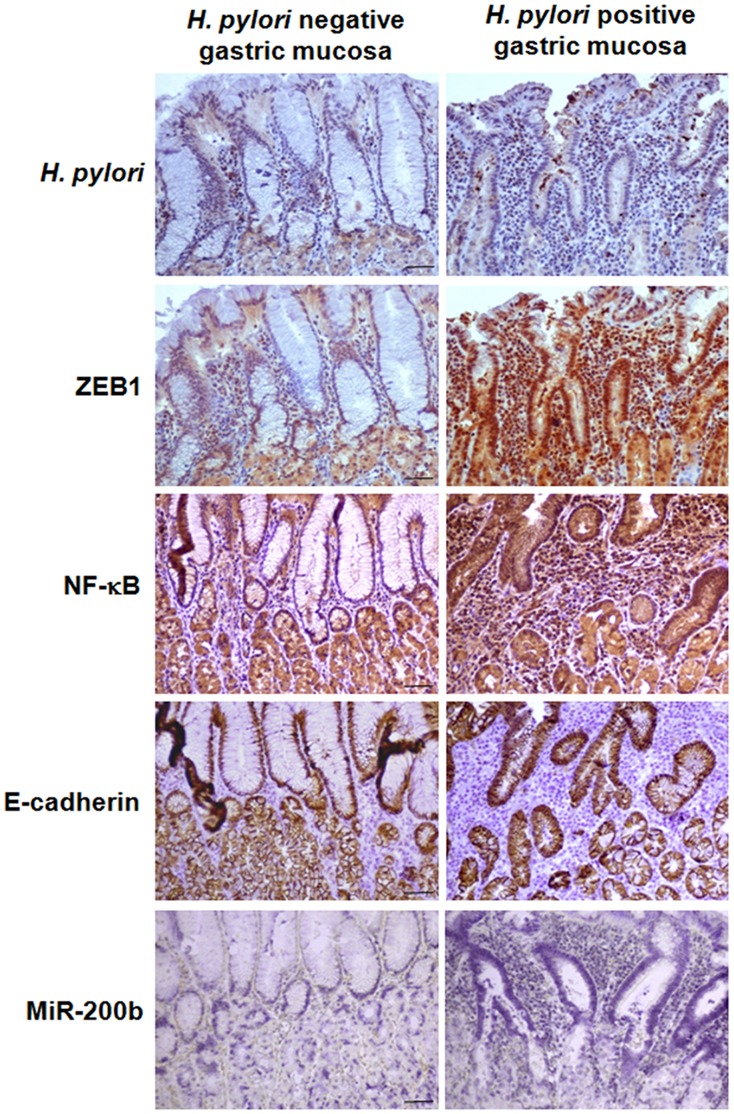
*H. pylori*, ZEB1, p65 NF-κB, E-cadherin immunostaining and miR-200b *in situ* hybridization in non infected human gastric mucosa (left pannels) or mucosa infected with *cag*PAI+ *H. pylori* (right pannels). Images are representatives of the detection by immunohistochemistry coupled to peroxydase activity (in brown) of *H. pylori* in the lumen of gastric glands at the apical surface of gastric epithelial cells, which display an intense ZEB1 and p65 expression mainly in the nucleus, despite a similar E-cadherin expression and localization at cell/cell junction in the *H. pylori* infected specimen and the non-infected one. MiR-200b detected by ISH coupled to phosphatase alkaline activity (in dark blue) is highly expressed in gastric glands of *H. pylori*–infected case. Typical images of the same case out of three infected patients and the same case out of three uninfected cases are shown. Bar, 50 µm.

To evaluate the *in vivo* relevance of these findings, ZEB1, miR-200b, E-cadherin expressions and the activation of NFκB were investigated on gastric mucosa tissue sections from patients infected with *H. pylori* (all with *cag*PAI positive strains, n = 3) or uninfected (n = 3). As compared to uninfected gastric mucosa (Fig. 5, left panels), the *H. pylori*-infected mucosa is characterized by a massive inflammation within the mucosa associated to an altered architecture of the glandular epithelium, which appeared atrophic compared to non-infected one (Fig. 5, right panels). The cell polarity in the epithelium, characterized by the position of the nucleus at the basal pole, is nevertheless conserved. In the upper part of the gastric epithelium, both ZEB1 and NFκB nuclear immunostaining are observed in human gastric glands colonized by *cag*PAI*+ H. pylori* strains, but not in uninfected mucosa. E-cadherin is detected at the cell membrane between adjacent epithelial cells in either infected or non-infected mucosa, indicating that cell-to-cell junctions remained integral despite the morphological changes of the glandular epithelium observed in the infected mucosa compared to the non-infected one. MiR-200b, revealed by *in situ* hybridization, is accumulated at higher levels in the cytoplasm and in the nuclei at the basal pole of the gastric epithelial cells of the infected mucosa, compared to the uninfected one. These histological observations of simultaneous NFκB activation and ZEB1, E-cadherin, miR-200 expressions in areas of *H. pylori* colonization and associated chronic inflammation corroborate our *in vitro* data on gastric cell lines.

## Discussion

Many, if not all, of the pathologies linked to *H. pylori* are caused by its ability to induce a chronic gastritis in the human stomach. The severity of this inflammation is associated to the bacterial *cag*PAI and caused by the persistent production of chemokines and cytokines under the constant activation of NF-κB by the pathogen detection systems of both gastric epithelial and infiltrated immune cells. Besides its key role in the mediation of innate and adaptive immunity, NF-κB also mediates EMT, a process prominently involved in cell migration in embryogenesis, wound healing and cancer progression [26]; [27]. *H. pylori* infection is the major risk factor in gastric carcinogenesis, mainly through the *cag*PAI-encoded CagA effector. The typical hummingbird phenotype acquired by AGS cell line *in vitro* reports the morphogenic activity of CagA upon its interaction with SHP-2 phosphatase and microtubule-associated PAR1 [8]; [12]. We show here that these EMT-like morphological changes, specifically induced by *cag*PAI+ *H. pylori* in gastric epithelial cells, are associated to enhanced expression of mesenchymal genes and are regulated by a tripartite NF-κB/ZEB1/miR-200 signaling pathway.

EMT is not obvious *in vivo* at a morphological point of view ([28] and our observations in Fig. 5), but in tissue culture and depending on cell systems, it is characterized by the loss of cell polarity and cell-cell interactions, modulation of cell matrix adhesion, enhanced extracellular matrix proteolysis, reorganization of the cytoskeleton, and acquisition of cell motility [15]. Despite their epithelial aspect, the gastric cell lines herein used were not polarized in our growth conditions. Nevertheless, cell polarity has been shown affected by CagA through its interaction with PAR-1, a member of the conserved PAR family regulating cell polarization [12]. Corroborating others’ data [9]; [29], our observations clearly show diverse manifestations of loss of cell-cell interactions in AGS or MKN74 cells challenged with *cag*PAI*+ H. pylori,* likely involving reorganization of the cytoskeleton. At last CagA promoted cell motility [11]. At a genetic point of view, several transcription factors have been characterized in EMT: the Snail superfamily of zinc-finger factors, the ZEB family, and basic helix-loop-helix factors. These factors down-regulate E-cadherin and up-regulate Vimentin, MMP9, and integrin α5. Several of these mesenchymal markers and transcription factors are up-regulated by wt *H. pylori* in the gastric epithelial cell lines herein used. Notably, the key EMT-promoter ZEB1 appears as a major contributor in *H. pylori* morphogenic activity, as its knockdown specifically prevents the mesenchymal transition of infected cells. ZEB1 up-regulation in the infected gastric cell lines depends on *H. pylori cag*PAI, and belongs to early events occurring during the first hours upon addition of bacteria to epithelial cell cultures. Similarly, ZEB1 is clearly detectable in epithelial cells of *cag*PAI*+ H. pylori*-infected human gastric mucosa prior to their neoplasic transformation. So far ZEB1 has been studied rather in the context of cancer than that of infection. Its expression in breast, prostate and colorectal cancers constitutes a poor prognostic factor [18]; [30]; [31]. In gastric carcinoma, ZEB1 expression is correlated with metastasis, corroborating its relevance in gastric cancer progression [32]. In a breast cancer cell line, ZEB1 expression is dependent on NF-κB, which is constitutively activated in cancer and stromal cells of breast cancers, and promotes EMT and cell survival [25]. Here, we confirm the NF-κB-dependent ZEB1 up-regulation in the *H. pylori-*infected gastric AGS cell line by ChIP and IκB transfection assays. In addition, infected gastric glands, which strongly express ZEB1, show evidence of NF-κB activation with its nuclear location, compared to uninfected mucosa. In addition, EMT effectors including vimentin, MMP9 or Snail1, that we find up-regulated in the infected gastric cell lines, have also been reported to be transactivated by NF-κB [33]; [34]; [27]. Therefore, they all may participate with ZEB1 to *H. pylori* morphogenic effects. Of note, the transcriptional activity of Snail1 has recently been reported to be dependent on LATS2 [35]. We precisely found that LATS2 up-regulation in infected AGS cells controls their cell cycle arrest [24]. This suggests that this cell cycle regulating kinase could likewise potentiate *H. pylori* morphogenic effects.

Enhanced expression of *snail*, *twist, vimentin* have been reported in human gastric cell lines infected by *H. pylori* and in a gastric carcinoma mouse model [36]. In canine MDCK cells, which switch from a polarized to invasive phenotypes upon CagA stimulation, only vimentin is up-regulated [37]. This suggests that the full *H. pylori cag*PAI may be requiered for ZEB1 or Snail up-regulation, likely by eliciting higher levels of activated NF-κB than CagA alone. Indeed, the PG translocated *via* the T4SS also stimulates IκB kinase [4]. Moreover, the pro-inflammatory cytokines themselves may sustain EMT [38].

ZEB1/2 are post-transcriptionally regulated by miR-200s and related to this miRNA family by a double negative feedback loop [16]; [20]. Accordingly, miR-200s predominate in epithelial cells and prevent ZEB expression, allowing epithelial genes to be fully expressed, while in mesenchymal cells ZEB1/2 prevent miR-200s and epithelial gene expressions. In basal conditions, AGS, MKN-74 and NCI-N87 cell lines perfectly fit to this model, since EMT and ZEB1 up-regulation can be achieved solely by manipulation of miR-200b/c levels using antisense oligonucleotides, as in other epithelial cell lines [22]; [23]. However, in *H. pylori-*infected conditions, miR-200b/c are increased despite ZEB1 accumulation in the gastric cell lines and their mesenchymal transition. MiR-200b/c up-regulation was also observed in our previous comparative deep-sequencing data on the miRNome of AGS co-cultured with or without *H. pylori* [24]. We identified a mechanism that partly could explain the paradoxical miR-200 up-regulation in infected cells: analyzing *miR-200b-a-429* promoter, we unveiled a NF-κB binding site stimulating promoter activity and pri-miR-200b-200a-429 synthesis, and leading to higher miR-200b levels. The NF-κB binding site overlaps one of the ZEB1-binding E-boxes, suggesting that under certain circumstances and according to their respective amounts, ZEB1 and NF-κB may compete for binding on the promoter. This is likely to occur in the co-culture experiments as soon as the first hours of infection and detection of pathogen-associated motives, as well in the inflamed gastric mucosa of chronically infected patients, in which concomitant NFκB activation and ZEB1 and miR-200b over-expressions can be observed. Moreover, this process is reversible in culture, in accordance to EMT definition, since the initial ZEB1 expression is restored after the bacteria have been removed. Nevertheless two out of the three cell lines keep stigmas of their previous infection and display decreased miR-200b/c levels compared to uninfected cells. Changes in miR-200 have been reported in pathological gastric mucosa as compared to non-pathological states. In accordance with our data, miR-200s are up-regulated in mouse models of chronic inflammation and gastritis-associated tumors [39]. Conversely, global miRNA analyses of *H. pylori*-infected human gastric mucosa revealed 30 miRNAs significantly decreased in the *H. pylori*-positive group, among which all the miR-200 family members [40]. These results concern the global gastric mucosa including several distinct cell types and cannot be attributed to gastric epithelial cells specifically.

*H. pylori*-induced E-cadherin up-regulation cannot either be explained by ZEB1 up-regulation, which, as *cdh1* transcriptional repressor, should have led instead to decreased E-cadherin mRNA. It may not directly be regulated by NF-κB activation, as we did not find any binding site in the *cdh1* promoter sequences, but likely by some other signaling pathway activated by *H. pylori*, such as the p38 MAPK one, as shown in other circumstances [41]. This epithelial marker is actually a CagA target in the c-Met/p120-catenin/E-cadherin complex in the host cells [11]. E-cadherin delocalization in infected NCI-N87 cells may attest for this CagA-targeted action. Recovery of a functional E-cadherin in the E-cadherin-deficient AGS cells abrogates their plasticity and invasion abilities upon *H. pylori* infection [11], suggesting that, as long as E-cadherin is maintained in the host cell membrane, as shown in the gastric mucosa of the infected patients, it may fulfill a role of “gatekeeper” of EMT. The E-cadherin up-regulation, which we observed here in the gastric cells lines undergoing an EMT initiation, may reflect this particular function.

In conclusion, our data clearly show that the inflammatory background supported by *cag*PAI*+ H. pylori* strains and mediated par NF-κB activation promotes ZEB1 expression and initiates a mesenchymal phenotype in gastric epithelial cells, in which numbers of mesenchymal genes are up-regulated, along with the epithelial markers E-cadherin and the miR-200s. It will be worth further exploring the precise mechanisms of transcriptional up-regulation of these epithelial genes during *H. pylori*-induced EMT-like initiation. An attractive hypothesis of the role of that regulation could be that enhanced *cdh1* and miR-200 gene expression in *H. pylori*-challenged epithelial cells could provide a feedback control that would keep in check the effects of excessive NF-κB-mediated ZEB1 production, impede EMT progression and allow the process to stay reversible. How this signaling network contributes to the neoplastic progression in chronically infected patients would deserve a special attention. EMT is thought to influence cell growth arrest, resistance to apoptosis and production of cancer stem cells [15]. Thereby, it may contribute to a reduced renewal of the infected gastric epithelium and aberrant differentiation, which leads to chronic atrophic gastritis and metaplasia [42]. Contrarily to the miR-200 levels, which appear to be more versatile, ZEB1 could constitute an early marker of these preneoplastic lesions in *cag*PAI*+ H. pylori*-infected mucosa.

## Supporting Information

Figure S1 **Cell morphology of AGS, MKN74 or NCI-N87 cells, not infected (NI) or upon 24 h infection with either** ***cagPAI+*** **wild type** ***H. pylori*** **(Hp WT) or its isogenic mutants deleted either for** ***cagA*** **(Hp ΔCagA) or** ***cagE*** **(Hp ΔCagE), each at MOI 100 bacteria/cell.** In Hp WT-infected AGS or NCI-N87 cells, cells with typical mesenchymal phenotype are highlighted. Hp WT-infected MKN-74 cell cultures release floating cell clusters in the supernatant medium (insert). Cells were observed by phase contrast microscopy on an inverted Zeiss Axiovert 200 microscope. Bar, 40 µm.
(DOCX)

Figure S2 **E-cadherin immunolabeling in NCI-N87 cells in basal conditions (left panel) or upon infection with wild type** ***H. pylori*** **at MOI100 (right panel).** Cell were fixed and labeled stepwise with a anti-E-cadherin monoclonal antibody (1/1,600 dilution, Sigma, France), and then with a mixture containing a AlexaFluor 488-labeled anti-mouse secondary antibody (1/2,000 dilution), AlexaFluor 546-labelled phalloidin (1/1,000 dilution) and 4′, 6-diamidino-2-phenyl-indole (DAPI, 1 µg/ml), all from Invitrogen (France), as described in Material and Methods. Images were acquired on a Leica confocal microscope.
(DOCX)

Figure S3 **Post-transcriptional regulation of ZEB1 expression by miR-200b&c.** (A) Inverse relationship between miR-200b&c and ZEB1 expressions in human cell lines. Upper panel, endogenous expression of mature miR-200b or-200c determined by RT-qPCR; bars indicate mean ± SD of miRNA expression normalized to U6 snRNA (n = 5). Lower panels, ZEB1 and tubulin immunoblots. (B) RTqPCR of miR-200b or -200c in AGS cells treated with 100 nM anti-200b/c (as200) or scrambled (sc200) oligonucleotides. Bars represent the mean ± SD of miRNA expression normalized to U6 snRNA and compared to non transfected (NT) cells (n = 3; *P<0.05; ***P<0.001). (C) Schematic representation of the psiControl vector and the psiZEB1 sensor containing the 3′ untranslated region (UTR) of the ZEB1 human gene, which harbors 5 and 3 predicted miR-200b and miR-200c, respectively, target sequences. The psi-ZEB1 sensor was obtained by cloning the ZEB1 3′UTRcDNA, retrieved by PCR from AGS genomic DNA and specific primers (Table S1), into the bicistronic psiCHECK-2 vector (Promega) between *XhoI* and *NotI* restriction sites downstream to *Renilla* luciferase gene. (D) ZEB1 translation efficiency assessed with the psiZEB1 sensor, and compared to that of psicontrol vector. Luciferase activities were measured 48 h post transfection. Bars indicate the mean ± SD of relative *Renilla* luciferase activity normalized to that of firefly and compared to psicontrol (n = 3; *P<0.05)
(DOCX)

Figure S4 **Up-regulation of miR-200b and miR-200c in MKN-74 and NCI-N87 cells 24 h post infection with** ***cagPAI+ H. pylori*** **(Hp WT) at MOI 100 bacteria/cell.** Bars represent mean ± SD of RTqPCR data for miR-200b or miR-200b relative to U6 snRNA and compared to non infected cells (NI); n = 4, *: p-value <0.05; **, p<0.01; ***, p<0.001.
(DOCX)

Figure S5 ***MiR-200b-200a-429*** **promoter activity.** (A) *MiR-200b-200a-429* promoter activity in HEK293, AGS or MKN-74 cells measured by the promoter luciferase reporter; upper panel, bars represent mean ± SD of the luciferase activity of the miRNA promoter relative to that of SV40 promoter reporter (n = 2); lower panel, ZEB1 and tubulin immunoblots. (B) SV40 promoter (pGL3-p) or *miR-200b-200a-429* promoter (pGL3prom200b) activities in MKN-74 cells upon 24 h infection with wt *H. pylori* at MOI 100; bars represent mean ± SD of luciferase activities of each reporter vector (n = 3; **P<0.01) (C) Schematic representation of putative transcription factor binding sites in the promoter sequence, showing the E-boxes (bold) and the overlapping NF-κB binding site (underlined). The nucleotides that have been mutated in Fig. 4C are indicated by arrows.
(DOCX)

Figure S6 **NF-κB immunofluorescence in AGS cells transfected with pEGFP (left panels) or pEGFP-IκB (right panels) in basal conditions or upon infection.** Cells were seeded in 8-well Labteck™ chambers and transfected with the expression vectors at 100 ng/well. Forty eight hrs post-transfection, cells were infected with wild type *H. pylori* at MOI 100. Six hrs later, cell were fixed and labeled stepwise with a goat anti-NF-κB antibody and then with a AlexaFluor564-labeled anti-goat IgG secondary antibody, as described in Material and Methods. Images were acquired on a Zeiss microscope equipped with epifluorescence.
(DOCX)

Table S1 **List of oligonucleotide primers.**
(DOCX)

Table S2 **Expression of the miR-200 family members in gastric epithelial cell lines.** (A) Levels of miR-200 in basal conditions: values represent mean ± SD of RT-qPCR data for each miRNA relative to snoR25 (n = 4). (B) Variations of miR-200a, -429 and -141, 24 h post-infection with *cag*PAI*+ H. pylori* (Hp WT) or the isogenic CagA-deficient strain, both at MOI 100. Data represent mean ± SD of RT-qPCR data for each miRNA relative to snoR25 and compared to non infected cells (NI); n = 4; *: p-value <0.05, ***: p-value <0.001.
(DOCX)

Material and Methods S1 (DOCX)
