# Supplementary figures and images for: Helicobacter pylori Initiates a Mesenchymal Transition through ZEB1 in Gastric Epithelial Cells
Source: PLoS One. 2013 Apr 2;8(4):e60315. doi: 10.1371/journal.pone.0060315 (PMC3614934; doi:10.1371/journal.pone.0060315)

**Figure S1**

**
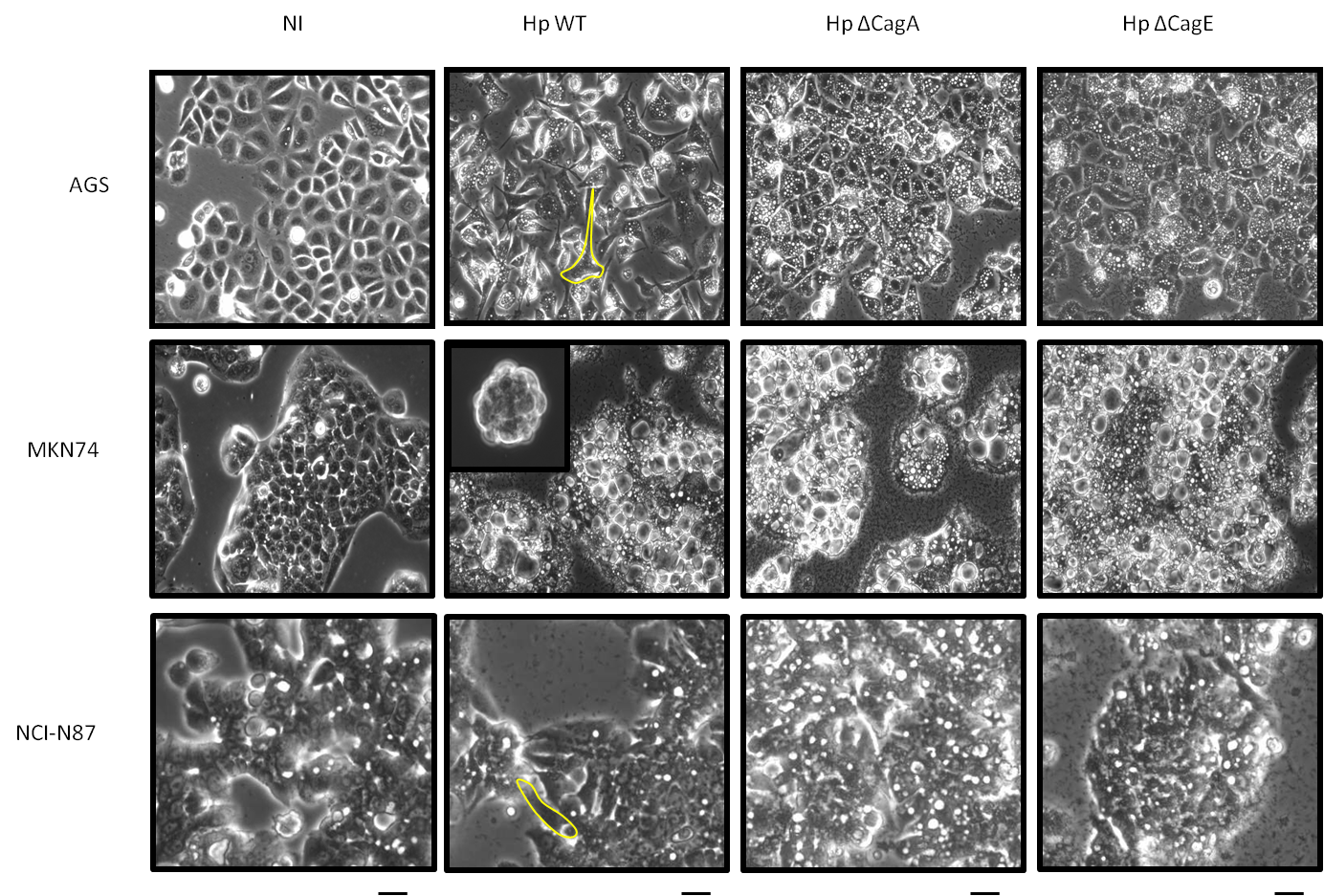
**

Supplement: Figure S1 — Cell morphology of AGS, MKN74 or NCI-N87 cells, not infected (NI) or upon 24 h infection with either cagPAI+ wild type H. pylori (Hp WT) or its isogenic mutants deleted either for cagA (Hp ΔCagA) or cagE (Hp ΔCagE), each at MOI 100 bacteria/cell. In Hp WT-infected AGS or NCI-N87 cells, cells with typical mesenchymal phenotype are highlighted. Hp WT-infected MKN-74 cell cultures release floating cell clusters in the supernatant medium (insert). Cells were observed by phase contrast microscopy on an inverted Zeiss Axiovert 200 microscope. Bar, 40 µm. (DOCX) [file pone.0060315.s001.docx]

**Figure S3**

**
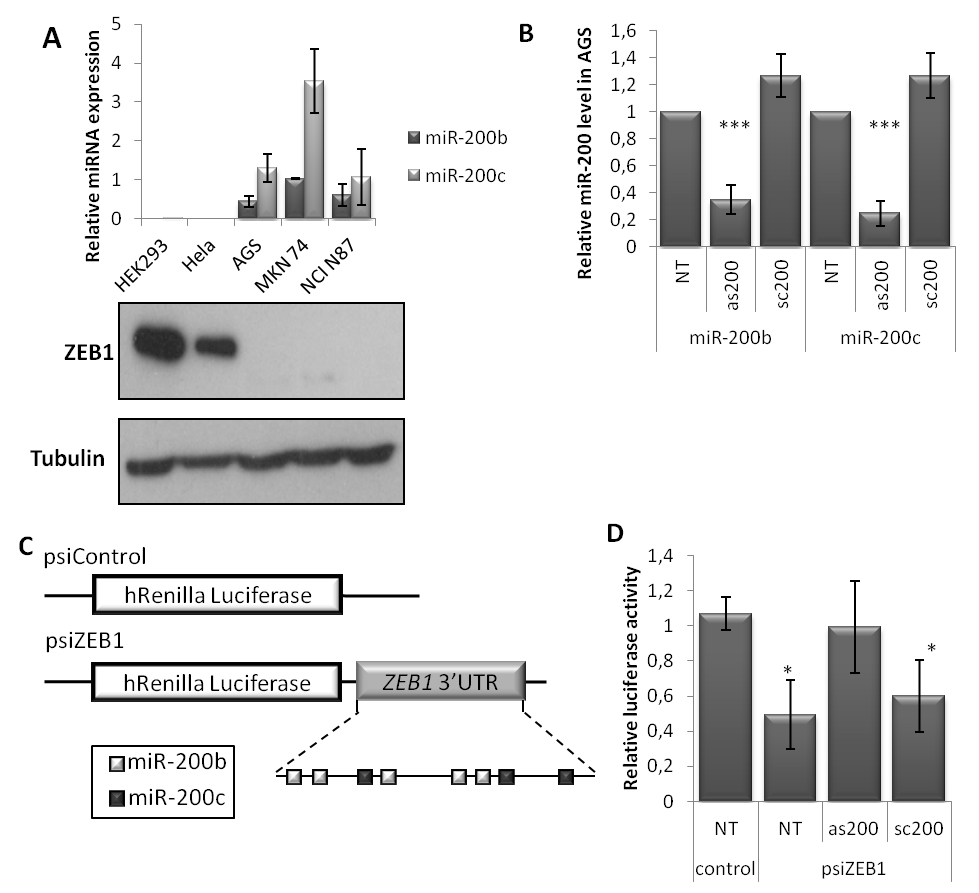
**

Supplement: Figure S3 — Post-transcriptional regulation of ZEB1 expression by miR-200b&c. (A) Inverse relationship between miR-200b&c and ZEB1 expressions in human cell lines. Upper panel, endogenous expression of mature miR-200b or-200c determined by RT-qPCR; bars indicate mean ± SD of miRNA expression normalized to U6 snRNA (n = 5). Lower panels, ZEB1 and tubulin immunoblots. (B) RTqPCR of miR-200b or -200c in AGS cells treated with 100 nM anti-200b/c (as200) or scrambled (sc200) oligonucleotides. Bars represent the mean ± SD of miRNA expression normalized to U6 snRNA and compared to non transfected (NT) cells (n = 3; *P<0.05; ***P<0.001). (C) Schematic representation of the psiControl vector and the psiZEB1 sensor containing the 3′ untranslated region (UTR) of the ZEB1 human gene, which harbors 5 and 3 predicted miR-200b and miR-200c, respectively, target sequences. The psi-ZEB1 sensor was obtained by cloning the ZEB1 3′UTRcDNA, retrieved by PCR from AGS genomic DNA and specific primers (Table S1), into the bicistronic psiCHECK-2 vector (Promega) between XhoI and NotI restriction sites downstream to Renilla luciferase gene. (D) ZEB1 translation efficiency assessed with the psiZEB1 sensor, and compared to that of psicontrol vector. Luciferase activities were measured 48 h post transfection. Bars indicate the mean ± SD of relative Renilla luciferase activity normalized to that of firefly and compared to psicontrol (n = 3; *P<0.05) (DOCX) [file pone.0060315.s003.docx]

**Figure S4**


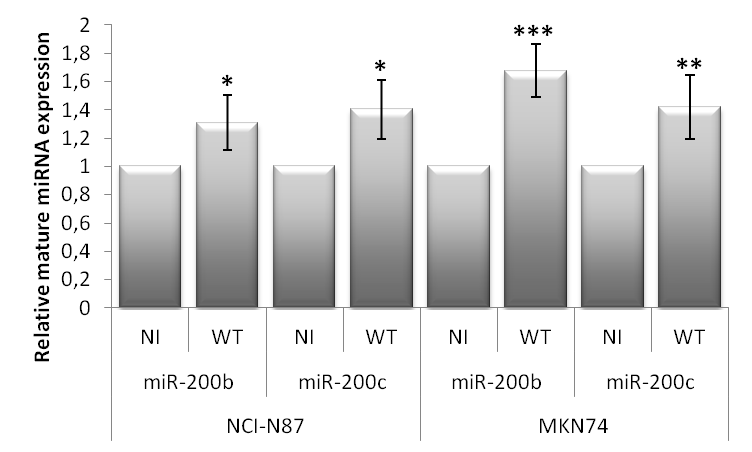

Supplement: Figure S4 — Up-regulation of miR-200b and miR-200c in MKN-74 and NCI-N87 cells 24 h post infection with cagPAI+ H. pylori (Hp WT) at MOI 100 bacteria/cell. Bars represent mean ± SD of RTqPCR data for miR-200b or miR-200b relative to U6 snRNA and compared to non infected cells (NI); n = 4, *: p-value <0.05; **, p<0.01; ***, p<0.001. (DOCX) [file pone.0060315.s004.docx]

**Figure S5**


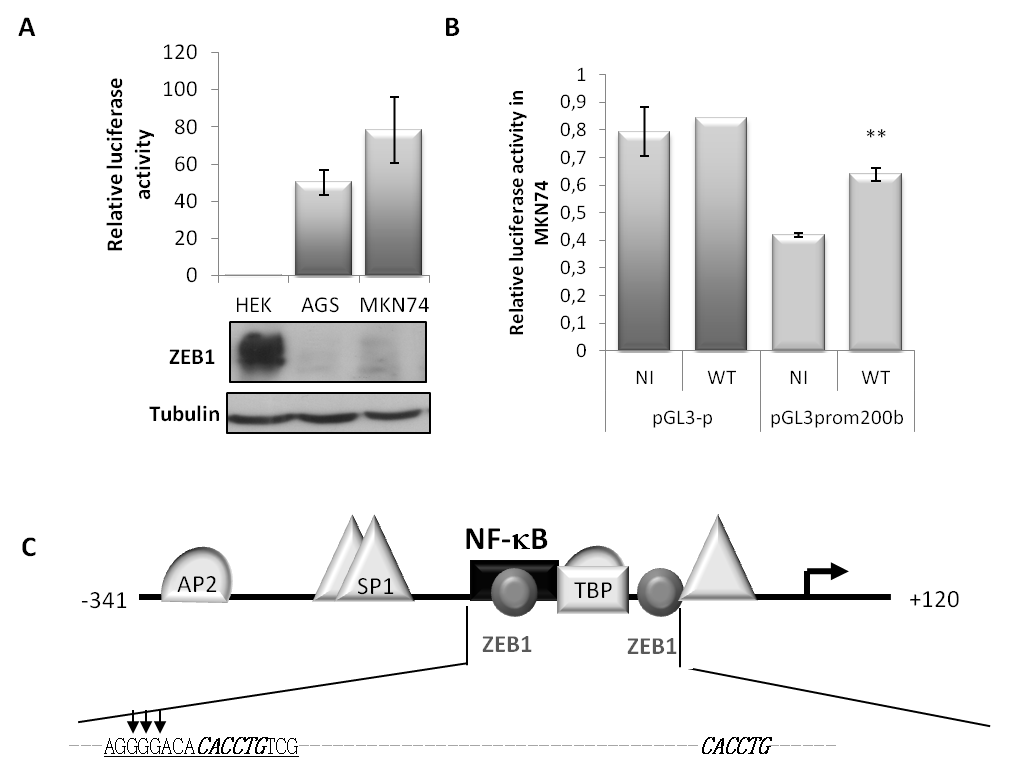

Supplement: Figure S5 — MiR-200b-200a-429 promoter activity. (A) MiR-200b-200a-429 promoter activity in HEK293, AGS or MKN-74 cells measured by the promoter luciferase reporter; upper panel, bars represent mean ± SD of the luciferase activity of the miRNA promoter relative to that of SV40 promoter reporter (n = 2); lower panel, ZEB1 and tubulin immunoblots. (B) SV40 promoter (pGL3-p) or miR-200b-200a-429 promoter (pGL3prom200b) activities in MKN-74 cells upon 24 h infection with wt H. pylori at MOI 100; bars represent mean ± SD of luciferase activities of each reporter vector (n = 3; **P<0.01) (C) Schematic representation of putative transcription factor binding sites in the promoter sequence, showing the E-boxes (bold) and the overlapping NF-κB binding site (underlined). The nucleotides that have been mutated in Fig. 4C are indicated by arrows. (DOCX) [file pone.0060315.s005.docx]
