# Supplementary material for: Helicobacter pylori Initiates a Mesenchymal Transition through ZEB1 in Gastric Epithelial Cells
Source: PLoS One. 2013 Apr 2;8(4):e60315. doi: 10.1371/journal.pone.0060315 (PMC3614934; doi:10.1371/journal.pone.0060315)
Supplement: Table S1 — List of oligonucleotide primers. (DOCX) [file pone.0060315.s007.docx]

**Table S1**

| **Commercial primers for RT-qPCR** | | |
| --- | --- | --- |
| **Name** | **References** | **Suppliers** |
| Hs-BMP1-1-SG | QT00000819 | QIAGEN |
| Hs-MMP9-1-SG | QT00040040 | QIAGEN |
| Hs-SNAI3-1-SG | QT00213045 | QIAGEN |
| Hs-ITGA5-1-SG | QT00080871 | QIAGEN |
| Hs-KRT7-3-SG | QT01672951 | QIAGEN |
| Hs-SPP1-1-SG | QT01008798 | QIAGEN |
| *hsa-miR-200b* | AB 001800 Taqman | Applied Biosystems |
| *hsa-miR-200c* | AB 002300 Taqman | Applied Biosystems |
| RNU6b | AB 001903 Taqman | Applied Biosystems |

| **Cloning** | | |
| --- | --- | --- |
| **Name** | **Sequence** | **Tm (°C)** |
| ZEB1-3’UTR-Fwd | AAA-ACT-CGA-GCA-AAA-TAA-ATC-CGG-GTG-TGC* | 60 |
| ZEB1-3’UTR-Rev | AAA-AGC-GGC-CGC-TAC-AGT-ATT-ATA-CAC-TAC-AC** | 60 |
| Prom-200b-Fwd | AAA-AGG-TAC-CAG-GGG-TGG-AGA-GGC-GAG-AGT*** | 60 |
| Prom-200b-Rev | AAA-ACT-CGA-GCC-TGG-CAC-AGG-AAG-TCA-GTT* | 60 |
| P200bmutNFkB-F | GCC-TAT-GGG-AGC-CCA-AAA-GAC-ACA-CCT-GTC-GGG-GGC-CA | 68 |
| P200bmutNFkB-R | CCC-GAC-AGG-TGT-GTC-TTT-TGG-GCT-CCC-ATA-GGC-CGG-GC | 69 |

| **RT-qPCR** | | |
| --- | --- | --- |
| **Name** | **Sequence** | **Tm (°C)** |
| ZEB1-Fwd | AAG-AAA-GTG-TTA-CAG-ATG-CAG-CTG | 60 |
| ZEB1-Rev | CCC-TGG-TAA-CAC-TGT-CTG-GTC | 60 |
| ZEB2-Fwd | CAA-GAG-GCG-CAA-ACA-AGC | 60 |
| ZEB2-Rev | GGT-TGG-CAA-TAC-CGT-CAT-CC | 60 |
| Vimentine-Fwd | AGA-TGG-CCC-TTG-ACA-TTG-AG | 60 |
| Vimentine-Rev | CCA-GAG-GGA-GTG-AAT-CCA-GA | 60 |
| Twist-Fwd | TCC-GCG-TCC-CAC-TAC-CA | 56 |
| Twist-Rev | TTC-TCT-GGA-AAC-AAT-GAC-ATC-TAG-GT | 56 |
| Snail1-Fwd | GCT-GCA-GGA-CTC-TAA-TCC-AGA | 58 |
| Snail1-Rev | ATC-TCC-GGA-GGT-GGG-ATC | 58 |
| Slug-Fwd | TGG-TTG-CTT-CAA-GGA-CAC-AT | 58 |
| Slug-Rev | GTT-GCA-GTG-AGG-GCA-AGA-A | 58 |
| primiR-200b-Fwd | GCG-GTG-ATG-ATT-AAC-CCA-AC | 60 |
| primiR-200b-Rev | GTG-GCC-ACA-GGT-CAA-GAA-AT | 60 |
| Cdh1-Fwd | TGA-GTG-TCC-CCC-GGT-ATC-TTC | 60 |
| Cdh1-Rev | CAG-TAT-CAG-CCG-CTT-TCA-GAT-TTT | 60 |
| HPRT1-Fw | TGA-CAC-TGG-CAA-AAC-AAT-GCA | 60 |
| HPRT1-Rev | GGT-CCT-TTT-CAC-CAG-CAA-GCT | 60 |
