# Supplementary material for: Helicobacter pylori Initiates a Mesenchymal Transition through ZEB1 in Gastric Epithelial Cells
Source: PLoS One. 2013 Apr 2;8(4):e60315. doi: 10.1371/journal.pone.0060315 (PMC3614934; doi:10.1371/journal.pone.0060315)
Supplement: Table S2 — Expression of the miR-200 family members in gastric epithelial cell lines. (A) Levels of miR-200 in basal conditions: values represent mean ± SD of RT-qPCR data for each miRNA relative to snoR25 (n = 4). (B) Variations of miR-200a, -429 and -141, 24 h post-infection with cagPAI+ H. pylori (Hp WT) or the isogenic CagA-deficient strain, both at MOI 100. Data represent mean ± SD of RT-qPCR data for each miRNA relative to snoR25 and compared to non infected cells (NI); n = 4; *: p-value <0.05, ***: p-value <0.001. (DOCX) [file pone.0060315.s008.docx]

**Table S2**

| **A. Relative basal levels** | **Clustered on Chr 1** | | | **Clustered on Chr 12** | |
| --- | --- | --- | --- | --- | --- |
|  | **mir-200b** | **miR-200a** | **miR-429** | **miR-200c** | **miR-141** |
| **AGS** | 0.338 ± 0.030 | 0.0081 ±  0.0007 | 0.0053 ±  0.0003 | 0.453 ± 0.121 | 0.024 ±  0.002 |
| **NCI-N87** | 0.208 ± 0.030 | 0.0061 ±  0.0006 | 0.0057 ±  0.0006 | 0.448 ± 0.021 | 0.0068±  0.0006 |
| **MKN74** | 0.154 ± 0.004 | 0.0022 ±  0.0003 | 0.0059 ±  0.0005 | 0.428 **±** 0.064 | 0.0058 ±  0.0005 |

| **B. Fold regulation** | | **miR-200a** | **miR-429** | **miR-141** |
| --- | --- | --- | --- | --- |
| **AGS** | **Hp WT** | 1.152 ± 0.114 | 1.567 ± 0.064 (***) | 1.13 ± 0.068 |
|  | **Hp ΔCagA** | 0.937 ± 0.160 | 1.103 ± 0.123 | 1.055 ± 0.167 |
| **NCI-N87** | **Hp WT** | 1.64 ± 0.129 (***) | 1.159 ± 0.155 | 1.557 ± 0.310 (*) |
|  | **Hp ΔCagA** | 1.19 ± 0.09 | 0.906 ± 0.262 | 0.977 ± 0.051 |
| **MKN74** | **Hp WT** | 0.835 ± 0.116 | 1.196 ± 0.135 (*) | 1.527 ± 0.291 (*) |
|  | **Hp ΔCagA** | 1.082 ± 0.262 | 1.271 ± 0.164 (*) | 0.903 ± 0.144 |
