# Supplementary material for: Helicobacter pylori Initiates a Mesenchymal Transition through ZEB1 in Gastric Epithelial Cells
Source: PLoS One. 2013 Apr 2;8(4):e60315. doi: 10.1371/journal.pone.0060315 (PMC3614934; doi:10.1371/journal.pone.0060315)
Supplement: Material and Methods S1 — (DOCX) [file pone.0060315.s009.docx]

**SUPPLEMENTARY** **MATERIAL AND METHODS**

**RNA extraction**. Total RNA was extracted using Trizol reagent (Invitrogen). RNA concentrations were determined by spectrophotometry (NanoDrop Technologies). RNA quality was analyzed on a 2100 Bioanalyzer (Agilent Technologies).

**Quantitative RT-PCR.** Messenger RNA and pri-miR-200b-200a-149 levels were determined in 2 steps, using the Maxima^TM^ First Strand cDNA Synthesis Kit on 1 μg total RNA and Maxima^TM^ SyBr Green qPCR mix (Fermentas) with specific primers (listed in Table S1) for the PCR step. TaqMan® microRNA assays (Applied Biosystems) were used to quantify the mature miR-200b and miR-200c in 20 ng total RNA, according to the manufacturer’s instructions. Real time qPCR was performed on a RotorGene cycler (Qiagen). MiRNA levels were normalized to U6 snRNA. The levels of mRNA or pri-miR-200b-200a-149 were normalized to hypoxanthine phosphoribosyltransferase (HPRT) mRNA. Relative expressions were calculated using the comparative Ct method.

**Vector constructions.** The minimal *miR-200b-200a-149* promoter was obtained by PCR from AGS genomic DNA and specific primers (Table S1) and cloned into the basic pGL3 vector (Promega) between *XhoI* and *KpnI* restriction sites upstream of the firefly luciferase gene, to produce the pGL3-prom200b vector. The putative NF-κB binding site on pGL3-prom200b was mutated by site-directed mutagenesis, using the primers indicated in Table S1. Each construct was produced according to standard procedures and verified by sequencing.

**Transfections and reporter assays.** All transfections were performed in 24-well plates using Lipofectamine 2000 (Invitrogen) according to the manufacturer’s protocol and previous data [1]. Cells were transfected three times with 100 nM of miR-200-antisense or scrambled DNA/LNA (locked nucleic acid) oligonucleotides (as200b: 5’-GT**C**AT**C**AT**T**AC**C**AG**G**CA**G**TA**T**TA-3’; as200c: 5’-TC**C**AT**C**AT**T**AC**C**CG**G**CA**G**TA**T**TA-3’; sc200: 5’-CT**A**CC**T**TA**A**TC**C**GC**C**GG**A**AT**T**TA-3’; in bold, the LNA positions). The expression vectors pEGFP-C3 or pEGFP-IκB, and the bicistronic psi-ZEB1 or the control psiCHEK-2 reporter vectors were transfected at 100 ng/well. The monocistronic firefly luciferase reporters pGL3-prom200b or the control pGL3-p, and NFκB-luciferase or the control TAL-luciferase (Beckton-Dickinson) were co-transfected at 100 ng/well in the presence of 3 ng/well pRL-SV40 vector. Firefly and *Renilla* luciferases were measured at 48 h post-transfection using the Dual Luciferase Assay (Promega). Results are expressed as *Renilla* luciferase activity relative to that of firefly for psi-ZEB1 and psiCHEK-2 reporters, and as firefly luciferase activity relative to that of *Renilla* for pGL3-prom200b, pGL3-p, NFκB-luciferase or TAL-luciferase reporters.

**Chromatin immunoprecipitation assays:** 24h post infection with *H. pylori* (MOI 100), non infected and infected cell layers were crosslinked with 1% formaldehyde in the culture medium at room temperature for 10 min. The following steps were performed according to Braken et al., except that cell lysis was extended to 20 min under agitation at 4°C and chromatin was sonicated for 15 min with cycles of 30 sec ON/30sec OFF [2]. Immunoprecipation was performed for 3h at 4°C under agitation with proteinA-sepharose beads coupled to 4 μg anti-NF-κB antibody (SantaCruz) or not coupled (control). The immune complex was recovered by centrifugation, washed and eluted [2]. Crosslinks was reversed by Proteinase K (160 μg/ml) and RNAse A (80 μg/ml) and DNA extracted by phenol/chloroform and ethanol precipitation. qPCR analysis was carried out with Maxima^TM^ SyBr Green PCR mix (Fermentas) and primers for the different promoters listed in Table S1.

**Human gastric tissue immunohistochemistry (IHC).** For histology processing, fresh tissue samples were fixed for 24 h in 3.7 % neutral-buffered formalin (Sigma), followed by standard histological processing and paraffin embedding. Sections of 3 µm thickness from paraffin-embedded tissues were used for standard IHC protocols with anti-ZEB1 (Bethyl; 1/100 dilution), anti-E-cadherin (Beckton-Dickinson France; 1/100 dilution), anti-NFκB p65 (Tebu France; 1/100 dilution) or anti-*H. pylori* (DAKO; 1/100 dilution) antibodies for 2h at room temperature, and then with labelled Polymer-Horse Radish Peroxidase anti-rabbit EnVision System (DAKO) for 30 min at room temperature. Immunolabeling was revealed by 10 min incubation in liquid diaminobenzidine-chromogen substrate (DAKO) at room temperature. Slides were counterstained with hematoxylin, dehydrated and mounted with Eukitt-mounting medium (Labonord).

***In situ* hybridization of miR-200b.** De-paraffinized and rehydrated tissue sections of 5 μm thickness were digested by Proteinase K (Invitrogen, 10μg/mL in Tris 50mM pH 7.6) for 15 min at room temperature, and post-fixed in 4% paraformaldehyde in PBS for 10 min. After 3 washes in PBS and one in SSC buffer 4x, the tissues were prehybridized for 2 hrs at 52 °C in formamide 50%, SSC 4x, dextran sulfate 200 mg/mL, tRNA 500 μg/mL, and hybridized overnight at 52°C in the same buffer containing 25 nM miR-200b DNA/LNA antisense (sequence before mentioned) labeled at both 3’ and 5’ ends with digoxigenin. After 2 stringent washes in SSC 0.2x at 52°C, the hybridized antisense was revealed with an anti-digoxigenin antibody coupled to alkaline phosphatase (Roche, 1/400 dilution, overnight incubation at 8°C), followed by an incubation for 2 hrs at room temperature in a 1-Step™NBT/BCIP plus Suppressor solution (Pierce). Slides were dehydrated and mounted with Eukitt-mounting medium.

***Helicobacter* detection.** *H. pylori* was detected in fresh aseptically collected human gastric mucosa by direct culture of the bacteria as previously described.[3] *H. pylori* growth was confirmed morphologically by phase contrast microscopy and Gram staining and biochemically by urease, catalase, and oxidase reactions. Genomic DNA was extracted from *H. pylori* cultures for PCR amplification and sequencing of *Helicobacter* 16S rDNA, *cag*A, and *cag*E [3].

**REFERENCES**

[1] Belair C, Baud J, Chabas S, Sharma CM, Vogel J et al. (2011) Helicobacter pylori interferes with an embryonic stem cell miRNA cluster to block cell cycle progression. Silence 2: 7-22.

[2] Bracken CP, Gregory PA, Kolesnikoff N, Bert AG, Wang J et al. (2008) A double-negative feedback loop between ZEB1-SIP1 and the microRNA-200 family regulates epithelial-mesenchymal transition. Cancer Res 68: 7846-54.

[3] Varon C, Dubus P, Mazurier F, Asencio C, Chambonnier L et al. (2012) Helicobacter pylori infection recruits bone marrow-derived cells that participate in gastric preneoplasia in mice. Gastroenterology 142 : 281-91.
